# Impact of an educational intervention on water, sanitation and hygiene knowledge, attitudes, and practices in early childhood development centres in low-socio-economic areas in the Nelson Mandela Bay, South Africa

**DOI:** 10.1371/journal.pone.0303077

**Published:** 2024-05-29

**Authors:** Paula Ezinne Melariri, June Teare, Opeoluwa Oyedele, Kirstie Eastwood, Wilma ten Ham-Baloyi

**Affiliations:** 1 Department of Environmental Health, Faculty of Health Sciences, Nelson Mandela University, Gqeberha, South Africa; 2 Department of Computing, Mathematical and Statistical Sciences, School of Science, University of Namibia, Windhoek, Namibia; 3 Statistical Consultation Unit, Nelson Mandela University, Gqeberha, South Africa; 4 Department of Nursing Science, Nelson Mandela University, Gqeberha, South Africa; Gadjah Mada University Faculty of Medicine, Public Health, and Nursing: Universitas Gadjah Mada Fakultas Kedokteran Kesehatan Masyarakat dan Keperawatan, INDONESIA

## Abstract

Good water, sanitation, and hygiene (WASH) enhance healthy living and safe environments for child development. The study aimed to evaluate the impact of an educational intervention on WASH status, knowledge, attitudes and practices in early child development (ECD) centres in low socio-economic areas in the Nelson Mandela Bay in 2021. This quasi-experimental, one group, pre-post-test study elicited responses from 51 ECD practitioners (1 per ECD centre). Telephonic structured knowledge, attitude and practices (KAP) questionnaires were used. KAP was good among participants. The educational intervention significantly improved mean knowledge (p<0.001, 95% CI: 0.58–1.11) attitudes (p<0.001, 95% CI: 0.39–0.67) and practices (p = 0.001, 95% CI: 0.20–0.74). WASH knowledge was significantly impacted by toilet facilities ventilation status (p = 0.083) while WASH attitudes scores were significantly impacted by ventilation where the potties are kept (p = 0.041). WASH practice scores were significantly impacted by across the bush/field (no facility) (p = 0.021) and plastic potties usage (p = 0.057). The educational intervention significantly improved WASH-related knowledge, attitudes, and practices among ECD practitioners. WASH conditions in the ECD centres in the study area require additional interventions targeted to sustainable strategies to enhance behavioural modifications for acceptability and sustainability of intervention strategies.

## Introduction

Water, sanitation, and hygiene (WASH) are vital indicators to healthy living and safe environments for child development. Poor sanitary conditions and unhygienic practices encourage the transmission of diseases such as diarrhoea and schistosomiasis, and are closely associated with the transmission of COVID-19 [1, 2]. Billions of people around the world do not have access to adequate sanitation and millions lack access to safe drinking water [3]. Safe drinking water, sanitation, and hygiene are crucial to good health, a safe environment, human dignity, an improved standard of living, and better educational outcomes [4]. The global need for access to safe drinking water and sanitation is emphasized in the Sustainable Development Goals (SDGs) programme for achievement by 2030 [5]. While substantial progress has been made towards achieving universal access to basic water, sanitation and hygiene, there are vast discrepancies in the quality of the services provided [6].

In developing countries, practices such as poor sanitation and hygiene play major roles in the spread of communicable diseases. For example, WASH baseline studies in Africa have shown that water sources are often at some distance from the community resulting in a high occurrence of container water storage (e.g., jerry cans) [7]. Poor water storage practices along with limited hand washing practices (using both soap and water) have resulted in a high prevalence of diarrhoeal disease [7–9].

The incidence of diarrhoea among children aged 5 years and younger is used as an indicator of the health of a community as diarrhoea has a more profound effect on children at the early years of life and the elderly [10]. Diarrhoea accounts for 46% of deaths on the African continent and 16% in South Africa in children younger than 5 years of age [11, 12]. Data from UNICEF projects [13], a retrospective study [14] and a systematic review [15] on hand washing practices in children in low- and middle-income settings confirmed that proper hand washing reduces the likelihood of gastrointestinal and respiratory diseases between 30% and 20%.With the advent of the COVID-19 pandemic, hand washing was reported as a key to preventing the spread of the virus [1, 16].

Caregivers and educators are uniquely placed to influence the hand washing practices of children [17]. Children are more likely to practice healthy behaviours at a young age; good hygiene behaviours, learnt at school level, are skills likely to be perpetuated as adults and passed on to their own children, provided suitable water, sanitation and hygiene-enabling facilities are present [8, 18].

Data from a systematic review [15] and randomised controlled trial [19] on WASH interventions have shown that the most effective interventions to reduce diarrhoea morbidity in children younger than 5 years of age are hand washing with soap, improved sanitation, and point-of-use water treatment. Hand washing with soap is the most cost-effective intervention and when applied at critical times such as before meal preparation and eating, and after toilet use, reduces diarrhoea rates [20]. Hand washing with soap can reduce incidences of acute respiratory infections and pneumonia, control of pandemic respiratory infection outbreaks [21], and reduce absenteeism among primary school children [22].

Water, sanitation and hygiene (WASH) programmes seek to estimate the availability of water, sanitation and hygiene; evaluate their use and effectiveness as well as devise and implement interventions in order to achieve these goals globally, nationally or at community level [23]. Furthermore, interventions aimed at improving teacher and learner knowledge, attitudes and practices through education, training programmes and health promotion at school level have been useful in preventing the spread of disease and improving the quality of life [11].

Despite the advancement made concerning water and sanitation, a vast number of areas in South Africa still lack adequate sanitation facilities and safe hygienic practices. A cross-sectional survey carried out in 2017 elicited responses from 46 early childhood development (ECD) centres which had a total of 3,254 children and 172 caregivers [24]. The aforementioned study evaluated the WASH in schools’ indicators (WinS) and findings showed that WinS for hygiene was poor despite the availability of improved water sources in 91% of the facilities, while the use of the bucket system was noted in 22% of the ECD centres. In 79% of the ECD centres, children washed their hands in a communal plastic bowl, while only 11% of the study population washed their hands hygienically under a running tap or tippy tap [24]. WASH conditions in the ECD centres in the previous study reported on the need for an urgent attention and further investigation for practical solutions. Previous reports from Cochrane reviews indicated four categories for WASH intervention measures [25, 26]. Emphasis was laid on: 1. Water quantity or supply improvement; 2. Measures geared towards enhancing water quality via removal or inactivating microbiological pathogens; 3. Interventions to enhance sanitation including promoting enhanced excreta disposal; and 4. Interventions to enhance hygiene aimed at initiating or promoting the practice of hand washing with soap or other agents after activities such as defecation, disposal of faeces, and to emphasise the need to wash hands adequately prior to preparing, eating and handling food items. Such interventions provided soap, hand washing stations, ‘Tippy Taps’ or other agents to participants or participating institutions geared towards promoting hygiene. However, at the time of study in 2021, it remained unclear what the WASH status and KAP were among ECD practitioners in ECD centres in the low-socio-economic areas of Nelson Mandela Bay, South Africa. Thus, this current study evaluated the impact of an educational intervention on the WASH status and KAP of ECD centres in the low-socio-economic areas of the Nelson Mandela Bay.

## Materials and methods

A quasi-experimental, one group, pre-post-test study design was used to evaluate the impact of an educational intervention on WASH status, knowledge, attitudes, and practices in ECD centres in low socio-economic areas in Nelson Mandela Bay.

The study was conducted in the Nelson Mandela Bay area in the Eastern Cape Province, South Africa. Study sites were selected based on socio-economic status and availability as well as accessibility of ECD centres in the area. The study sites were low-income residential/ commercial areas (including Motherwell Township, Walmer Township, New Brighton, Joe Slovo, Greenbushes and Zwide Township), and were situated in and around the city of Gqeberha (previously known as Port Elizabeth) which is the largest town in the Nelson Mandela Bay area and houses one of the country’s largest seaports. In addition, the study area is still under severe economic pressure, with unemployment rates close to 40% [27].

The registered number of ECD centres within the study area was 341 [28]. The selected sample consisted of one purposively selected ECD practitioner per ECD centre, based on the following criteria: (i) 19–60 years of age and (ii) who have consented to participate in the study. ECD centres were conveniently selected based on their socio-economic status, availability, willingness to participate as well as their proximity to other participants for logistic and budgetary reasons.

The sample size required for this study was calculated using the Slovin’s formula, *n = N*/(1+*Ne*^2^), for determining the sample size in a survey, where *n* is the sample size, *N* is the size of the population study and *e* is the margin of error. With a population of 341 ECD centres hence 341 practitioners (one practitioner per ECD centre) and 10% margin of error, the formula equates to a minimum sample size of n = 341/ (1+341×〖0.1〗^2) ≅78. Out of the 78 participants approached, only 51 consented to participate and completed the pre- and post-intervention assessments in the study.

The structured questionnaires were developed based on an existing questionnaire used in a similar WASH study on sanitation and hygiene in early childhood development [24] which adapted the WASH in schools’ (WinS) monitoring package [29] and the updated service ladders [30] in generating the questionnaire for this study, and pilot tested in May 2021 with six caregivers in six ECDs. No changes were required, and the results were included in the main study. Cronbach’s alpha coefficient—measuring the internal consistency, or reliability, of a set of survey items—scores indicated a level of quite reliable with scores of 0.6 for knowledge, 0.6 for attitudes and 0.8 for practices. Questionnaires were administered in English by trained fieldworkers telephonically to a principal or caregiver at each selected ECD centre. Collected data included the demographic characteristics of ECD centres, WASH status, and WASH knowledge, attitude, and practices (KAP). Responses from ECD practitioners to questions on the KAP questionnaire were entered into hand-held digital devices, supported by QuestionPro® (https://www.questionpro.com) data management system. Completion of the questionnaire took approximately 20 minutes. Data collection for the pre-intervention was conducted over a 3-month period (May-July 2021).

The intervention included a hard copy a WASH workbook in English with information about washing hands, germs, storing water safely, and the requirements for a safe and clean ECD centre. These were distributed by trained fieldworkers, independent to the study, to principals to be circulated to the ECD practitioners in the selected ECD centres. The WASH workbook was based on the worksheets produced in collaboration with UNICEF & Early Inspiration. The workbook provided participants with pictorial messages and illustrations for hand washing depicted in simple, clear and user-friendly format. By providing this information, it was expected that the WASH knowledge, attitudes and practices of ECD centres in the study area would be improved. The findings of the pre-test were used to tailor the intervention. Each participating ECD centre was also provided with a 25 litre heating bucket and a 5 litre bottle of dish washing liquid that can be used to facilitate hand washing and which remained at the ECD centres after the project had been completed. Two telephonic follow ups/monitoring were done with the principals of each of the ECD centres to check if the educational intervention was received and used by participants and whether any clarification regarding its use was required. To allow enough time, the intervention was implemented over a three-month period (August to October 2021) in the same way for all ECD centres. Thereafter, the data collection for the post-intervention was conducted, using the same approach used for the pre-intervention stage, from the end of October to November 2021.

Prior to data collection, the study received ethical approval from the Research Ethics Committee: Human (REC-H) of the Nelson Mandela University (H21-HEA-ENV-001) Permission was granted verbally from the principals of ECD centres. As approved by the ethics board, verbal informed consent was obtained from respondents to partake in the study, after explaining the study details, objectives and participant rights, and the questionnaire was anonymous.

### Data management and statistical analysis

The information obtained from the structured questionnaire was captured and analysed using Microsoft Office 365 (2019 version) and R software (version 4.2.1). The data cleaning and processing included checking the data for errors and the calculation of summated scores for the KAP towards WASH items in the questionnaire. Respondents were asked to respond to knowledge items as either true, false or unsure, with incorrect or unsure responses given a score of 0 and correct answers assigned a score of 1. The total score for knowledge ranged from 4 to 10 at the pre-intervention stage, and 6 to 10 at the post-intervention stage, with high scores indicating better knowledge of WASH. Knowledge items were further evaluated for internal reliability using the Cronbach’s alpha technique with an alpha value of 0.6 obtained, indicating internal reliability. For attitudes, respondents were asked to indicate the extent to which they agreed with some attitudinal statements on a Likert scale of 1 = strongly disagree, 2 = disagree, 3 = neutral, 4 = agree, and 5 = strongly agree. The scores were then calculated by averaging the respondents’ answers to the attitudinal statements, with total scores ranged from 3 to 5 at the pre-intervention stage and 3 to 4 at the post-intervention stage, with high scores indicating positive attitudes.

For the practice section, respondents were asked to rate their practice to items as either after each use, multiple times a day, once a day, every other day, weekly, less than weekly or not applicable. A score of 1 was given to answers that reflected good practice, while a score of 0 was given to answers that reflected bad practice. The total score pre-intervention ranged from 3 to 8 while at the post-intervention it ranged from 4 to 8, with high scores indicating better practices. Moreover, descriptive statistics and inferential statistics were used to analyse and describe the data, including the analysis of variance tests to assess the differences in mean values for the pre- and post-intervention WASH KAP scores. In addition, the analysis of variance tests were performed to assess the differences in the scores for KAP of WASH across the ECD centers’ characteristics. A p-value < 0.05 and 0.10 was considered significant.

## Results

### Response rate, demographic profile, and features of the ECD facilities

A total of n = 51 ECD practitioners participated in this study, resulting in a 65.4% response rate. As shown in Table 1, most of the centres (84.31%) recorded less than six practitioners and 64.71% of the centres hosted between 11 and 40 children. Facility type of the ECD centres varied from separate house (the most prevalent), to shipping container (the least prevalent). Structurally, the majority of ECD centres were permanent brick structures while some structures were built using corrugated iron or wood or a combination of bricks, corrugated iron, and wood. The majority of ECD centres had electricity and flush toilets and made use of plastic potties. However, the area where the potties were kept was poorly ventilated and in many instances (37.25%) the toilet facilities were used by both staff and children (Table 1).

**Table 1:** Demographic features of the ECD centres.

Variable	Responses	Count (n = 51)	%
**Number of Practitioners**	1–5	43	84.31
	6–10	5	9.80
	>10	3	5.88
**Number of children**	≥10	4	7.84
	11–40	33	64.71
	41–70	6	11.76
	71–100	7	13.73
	>100	1	1.96
**What is the type of facility (structure) of your centre?**	Apartment	4	7.84
	Separate house	21	41.18
	Part of caregiver’s house	8	15.69
	Wendy house	5	9.80
	Shipping container	1	1.96
	Other	12	23.53
**What main building materials have been used to construct the centre?**	Permanent bricks	30	58.82
	Temporary corrugated iron	5	9.80
	Temporary wood	3	5.88
	Combination of the above options	8	15.69
	Other	5	9.80
**Does the facility have electricity? **	Yes	45	88.24
	No	6	11.76
**Main source of drinking water for the centre**	Piped water into the centre	18	35.29
	Water tank	7	13.73
	Public tap or standpipe	23	45.10
	Protected spring	1	1.96
	Bottled water	1	1.96
	Tanker-truck	1	1.96
**Centre has flush toilet**	Yes	43	84.31
	No	8	15.69
**Centre has Ventilated Improved Pit latrine (VIP)**	Yes	4	7.84
	No	40	78.43
	Unsure	7	13.73
**Centre has bucket system**	Yes	10	19.61
	No	35	68.63
	Unsure	6	11.76
**Centre has no toilet facility (bush, field)**	Yes	1	1.96
	No	43	84.31
	Unsure	7	13.73
**Centre has plastic potties**	Yes	36	70.59
	No	12	23.53
	Unsure	3	5.88
**Are toilet facilities properly ventilated?**	Yes	40	78.43
	No	6	11.76
	Unsure	5	9.80
**Is the area where the potties are kept for use properly ventilated?**	Yes	37	72.55
	No	10	19.61
	Unsure	4	7.84
**Does the centre have separate toilet facilities for staff and children?**	Yes	32	62.75
	No	19	37.25

### WASH KAP characteristics

Tables 2–4 show the responses to items related to the KAP towards WASH. Participants recorded correct responses (≥80%) to each question on knowledge items post intervention (Table 2) Some of the items showed positive enhancement following the intervention. There was a notable improvement (13.73%) from participants’ responses post intervention on the item—‘Washing hands with water and soap is more effective than using disinfectant’. However, a few items recorded a 5.88% decline in the valid responses (Table 2).

**Table 2:** Responses to the questions on WASH knowledge.

	Pre-intervention (n = 51)				Post-intervention (n = 51)				Improvement correct scores post-pre (%)
	Incorrect answer		Correct answer		Incorrect answer		Correct answer		
Statement	Count	(%)	Count	(%)	Count	(%)	Count	(%)	
Can unsafe water cause diarrhoeal diseases?	3	5.88	48	94.12	6	11.76	45	88.24	-5.88
Can water get contaminated?	8	15.69	43	84.31	7	13.73	44	86.27	1.96
Was a clean water source used for hand washing?	3	5.88	48	94.12	1	1.96	50	98.04	3.92
Boiling water before consumption helps to remove disease causing microorganisms	1	1.96	50	98.04	0	0.00	51	100.00	1.96
Water containers must always be clean	0	0.00	51	100.00	3	5.88	48	94.12	-5.88
Waste can be breeding sites for flies and rodents	6	11.76	45	88.24	9	17.65	42	82.35	-5.88
Washing hands with water and soap is more effective than using disinfectant	15	29.41	36	70.59	8	15.69	43	84.31	13.73
The most crucial time to wash hands is before and after preparing meals	3	5.88	48	94.12	0	0.00	51	100.00	5.88
The most crucial time to wash hands is after using the toilet/bathroom	2	3.92	49	96.08	0	0.00	51	100.00	3.92
The most crucial time to wash hands is before eating	1	1.96	50	98.04	1	1.96	50	98.04	0.00

**Table 3:** Responses to the questions on WASH attitude.

	Pre-intervention (n = 51)				Post-intervention (n = 51)				Improvement correct scores: post-pre (%)
	Bad		Good		Bad		Good		Good scores
Statement	Count	(%)	Count	(%)	Count	(%)	Count	(%)	(%)
Children do not keep their hands clean after washing so there is no point†	9	17.65	42	82.35	12	23.53	39	76.47	-5.88
Caregivers should lead by example by keeping good hygiene	2	3.92	49	96.08	1	1.96	50	98.04	1.96
Hand washing is important	1	1.96	50	98.04	4	7.84	47	92.16	-5.88
Hand washing is hard†	42	82.35	9	17.65	40	78.43	11	21.57	3.92
I enjoy washing hands	3	5.88	48	94.12	8	15.69	43	84.31	-9.80
Washing hands every time further wastes the water that the Bay wants us to save†	23	45.10	28	54.90	12	23.53	39	76.47	21.57
It takes time to wash hands†	16	31.37	35	68.63	17	33.33	34	66.67	-1.96
I wash my hands to protect my own health	0	0.00	51	100.00	0	0.00	51	100.00	0.00
I wash my hands to protect the children’s health	0	0.00	51	100.00	0	0.00	51	100.00	0.00
Caregivers should be taught how to wash hands	5	9.80	46	90.20	3	5.88	48	94.12	3.92
I wash hands to clear my conscience	29	56.86	22	43.14	34	66.67	17	33.33	-9.80
It is the government’s responsibility to ensure the practices of safe hygiene and sanitation†	28	54.90	23	45.10	31	60.78	20	39.22	-5.88
It is the parents’ responsibility to ensure the practices of safe hygiene and sanitation†	46	90.20	5	9.80	47	92.16	4	7.84	-1.96

†Less than desirable responses given by respondents

**Table 4:** Responses to the questions on WASH practice.

	Pre-intervention (n = 51)				Post-intervention (n = 51)				Improvement good scores post-pre (%)
	Poor responses		Good responses		Poor responses		Good responses		
Statement	Count	(%)	Count	(%)	Count	(%)	Count	(%)	
How often are the toilets areas cleaned?	1	1.96	50	98.04	0	0.00	51	100.00	1.96
How often are the potties areas cleaned?	11	21.57	40	78.43	9	17.65	42	82.35	3.92
How often are the playrooms areas cleaned?	2	3.92	49	96.08	1	1.96	50	98.04	1.96
How often are the classrooms areas cleaned?	0	0.00	51	100.00	2	3.92	49	96.08	-3.92
How often are the food preparation areas cleaned?	1	1.96	50	98.04	0	0.00	51	100.00	1.96
Do you have a clearly demarcated and equipped nappy changing area?	7	13.73	44	86.27	4	7.84	47	92.16	5.88
Does the equipment used in this area have an easy to clean surface e. g. water-proof mattress?	9	17.65	42	82.35	6	11.76	45	88.24	5.88
Is the nappy changing area located in a separate room other than the food preparation area?	7	13.73	44	86.27	7	13.73	44	86.27	0.00

In terms of attitudes, minor improvements can be seen in the numbers of positive responses to valid comments after the intervention. However, most of the items such as—‘Caregivers should lead by example by keeping good hygiene’; ‘Hand washing is important’ recorded a significant decline post intervention. It was noted however that none of the participants at pre intervention indicated that washing hands was hard, however following the intervention 7.84% participants ‘strongly agreed’ that washing hands (possibly the ideal way) is hard (Table 3).

Furthermore, most responses to the practice items were good at both pre- and post-intervention stages and improved in the post-intervention stage except for ‘How often are the classrooms areas cleaned?’ which showed a decrease (Table 4). The mean WASH knowledge score was 9.2 (SD = 1.2, range: 4–10) with an overall accuracy rate of 92% (at 5% of significance) at pre-intervention stage, while for the post-intervention stage, it was 9.4 (SD = 0.9, range: 6–10) with an overall accuracy rate of 94%. The mean WASH attitude score was 3.5 (SD = 0.5, range: 3–5) at pre-intervention stage, and 3.5 (SD = 0.5, range: 3–4) post-intervention stage, indicating a positive, but no change in the attitude among the 51 respondents. Likewise, the mean score for WASH practices was 7.2 (SD = 1.3, range: 3–8) at pre-intervention stage, and 7.5 (SD = 1.0, range: 4–8) at post-intervention stage, indicating good practices among the respondents. The differences in mean values for the pre- and post-intervention scores for the KAP variables were assessed, and the results at 5% level of significance showed that mean differences between the pre- and post-intervention of WASH KAP were significant at p<0.001 for knowledge and attitude, and p = 0.001 for practices (Table 5).

**Table 5:** Comparison of the mean difference in WASH KAP scores.

	Diff	Std dev	T-value	Df	P-value	95% CI
Knowledge score	0.84	0.9	6.37	50	<0.001*	0.58–1.11
Attitude score	0.53	0.5	7.50	50	<0.001*	0.39–0.67
Practice score	0.47	0.9	3.55	50	0.001*	0.20–0.74

* Significant at 5% level of significance Diff–Difference

Std dev–Standard deviation Df–Degrees of freedom

CI–Confidence interval

### WASH KAP relative to ECD centres characteristics

The outputs obtained from assessing the differences in the scores for KAP of WASH across the ECD centres’ characteristics are shown in Table 6. Only the toilet facilities ventilation status (p = 0.083) characteristic showed a statistically different in the WASH knowledge scores at a 10% significance level for all the 51 respondents across their ECD centres’ characteristics. Regarding ventilation where the potties are kept, there was a statistically significant difference (p = 0.041) in the WASH attitudes scores at a 5% significant level. Furthermore, across the bush/field (no facility) (p = 0.021) and plastic potties usage (p = 0.057) characteristics, there were statistically significant differences in the WASH practice scores at a 5% and 10% level of significance, respectively (Table 6).

**Table 6:** Comparison of ECD centres characteristics and mean WASH KAP scores.

			Knowledge score			Attitude score			Practice score		
	Count (n = 51)	%	Mean	Std dev	P-value	Mean	Std dev	P-value	Mean	Std dev	P-value
**Number of caregivers**											
<10	46	90.20	9.20	8.90	0.877	23.00	4.24	0.149	9.20	12.89	0.173
≥10	5	9.80	1.40	0.89		2.50	2.12		1.60	2.61	
**Number of children**											
≤20	14	27.45	2.80	3.42	0.521	7.00	0.00	0.767	2.80	3.83	0.192
21–50	25	49.02	5.00	3.81		1.00	0.00		5.00	6.52	
51–80	7	13.73	1.40	2.19		3.50	2.12		1.40	3.13	
≥81	5	9.80	1.00	1.22		3.50	0.71		1.00	1.73	
**What is the type of facility (structure) of your centre?**											
Apartment	4	7.84	0.83	1.60	0.746	2.00	1.41	0.176	0.80	1.79	0.696
Separate house	21	41.18	3.83	3.82		10.50	7.37		4.20	6.10	
Part of caregiver’s house	8	15.69	1.83	1.47		4.00	2.08		1.60	2.51	
Wendy house	5	9.80	1.50	1.52		2.50	1.00		1.00	1.41	
Shipping container	1	1.96	1.00	2.00		0.50	2.65		0.20	0.45	
Other	12	23.53	3.00	2.90		6.00	2.00		2.40	3.91	
**What is the structure of the centre made of?**											
Permanent bricks	30	58.82	1.17	1.17	0.509	15.00	8.33	0.337	6.00	9.25	0.225
Temporary corrugated iron	5	9.80	1.00	1.26		2.50	0.58		1.00	1.41	
Temporary wood	3	5.88	2.00	1.67		1.50	1.73		0.60	0.89	
Combination of the above options	8	15.69	1.67	1.97		4.00	0.00		1.60	3.05	
Other	5	9.80	0.64	0.67		2.50	2.08		1.00	1.22	
**Does the facility have electricity?**											
Yes	45	88.24	7.67	8.31	0.979	22.50	12.42	0.483	9.00	13.73	0.936
No	6	11.76	1.33	1.21		3.00	1.15		1.20	1.64	
**Select the main source of drinking water for the centre**											
Piped water into the centre	18	35.29	3.17	4.58	0.755	9.00	4.62	0.132	3.60	6.43	0.609
Water tank	7	13.73	1.50	0.84		3.50	1.00		1.40	1.67	
Public tap or standpipe	23	45.10	4.33	3.50		11.50	4.93		4.60	6.50	
Protected spring	1	1.96	1.33	2.80		0.50	3.79		0.20	0.45	
Bottled water	1	1.96	1.83	4.02		0.50	5.51		0.20	0.45	
Tanker-truck	1	1.96	2.17	4.83		0.50	6.66		0.20	0.45	
**The main source of water**											
Mostly unreliable	2	3.92	0.67	1.03	0.817	1.00	0.58	0.471	0.40	0.89	0.486
Somewhat reliable	11	21.57	2.33	1.51		5.50	2.08		2.20	3.35	
Mostly reliable	7	13.73	1.83	1.83		3.50	0.58		1.40	2.61	
Always reliable	31	60.78	6.00	5.62		15.50	6.08		6.20	8.53	
**Where no running water is available, which type of low-cost hand washing points do you use?**											
Children wash hands in a plastic bowl with water	6	11.76	1.17	1.17	0.666	3.00	1.53	0.722	1.20	2.17	0.457
Children wash hands in a plastic bowl with detergents	3	5.88	0.83	0.98		1.50	0.58		0.60	0.89	
A pitcher of water and a basin (one person can pour the water for another to wash their hands)	5	9.80	1.33	1.21		2.50	0.58		1.00	1.73	
Tippy tap	19	37.25	3.83	3.60		9.50	4.04		3.80	5.81	
Children wash hands in a plastic bowl with soap	5	9.80	1.67	2.07		2.50	2.08		1.00	1.73	
Other	13	25.49	3.17	2.48		6.50	0.58		2.60	3.58	
**Flush toilet**											
Yes	43	84.31	7.33	8.16	0.614	21.50	11.93	0.859	8.60	12.78	0.368
No	8	15.69	1.67	1.21		4.00	1.15		1.60	2.51	
**Ventilated Improved Pit latrine (VIP)**											
Yes	4	7.84	0.83	0.98	0.641	2.00	1.15	0.120	0.80	1.30	0.896
No	40	78.43	7.00	6.99		20.00	10.58		8.00	11.55	
Unsure	7	13.73	1.67	1.63		3.50	1.53		1.40	2.19	
**Bucket**											
Yes	10	19.61	1.83	1.47	0.922	5.00	3.06	0.151	2.00	2.00	0.103
No	35	68.63	6.17	6.74		17.50	8.96		7.00	10.98	
Unsure	6	11.76	1.50	1.38		3.00	1.00		1.20	2.17	
**No facility (bush, field)**											
Yes	1	1.96	0.33	0.52	0.254	0.50	0.58	0.111	0.20	0.45	0.021*
No	43	84.31	7.50	8.14		21.50	11.53		8.60	12.58	
Unsure	7	13.73	1.67	1.21		3.50	1.53		1.40	2.61	
**Plastic potties**											
Yes	36	70.59	6.17	7.03	0.228	18.00	9.87	0.805	7.20	12.44	0.057**
No	12	23.53	2.33	2.34		6.00	2.52		2.40	2.07	
Unsure	3	5.88	1.00	1.26		1.50	1.00		0.60	0.89	
**Are toilet facilities properly ventilated?**											
Yes	40	78.43	6.83	8.16	0.083**	20.00	11.15	0.530	8.00	12.31	0.915
No	6	11.76	1.33	0.82		3.00	0.58		1.20	1.10	
Unsure	5	9.80	1.33	1.21		2.50	0.58		1.00	1.73	
**Is where the potties are kept for use properly ventilated?**											
Yes	37	72.55	6.33	7.28	0.104	18.50	10.69	0.041*	7.40	11.99	0.120
No	10	19.61	2.00	2.61		5.00	1.73		2.00	1.87	
Unsure	4	7.84	1.17	1.17		2.00	2.08		0.80	1.30	
**Does the centre have separate toilet facilities for staff and children?**											
Yes	32	62.75	5.50	6.66	0.129	16.00	8.72	0.269	6.40	10.11	0.354
No	19	37.25	3.50	3.27		9.50	5.00		3.80	4.97	
**Which of the following do children use for drinking water?**											
Individual water bottles	33	64.71	5.67	6.59	0.333	16.50	9.29	0.500	6.60	9.34	0.649
A water bucket with shared drinking cup	1	1.96	0.50	0.84		0.50	1.00		0.20	0.45	
A water bucket with individual drinking cups	13	25.49	2.67	1.86		6.50	2.08		2.60	4.72	
Other	4	7.84	1.33	1.75		2.00	1.15		0.80	1.30	
**Are you a registered Early Childhood Development Centre?**											
Yes	43	84.31	8.60	8.32	0.552	21.50	2.12	0.815	8.60	12.58	0.930
No	3	5.88	0.60	0.89		1.50	0.71		0.60	0.89	
Not sure	5	9.80	1.00	1.41		2.50	0.71		1.00	1.73	

* Significant at 5% level of significance

** Significant at 10% level of significance

## Discussion

This study aimed to evaluate the impact of an educational intervention on WASH status and knowledge, attitudes, and practices in ECD centres in low socio-economic areas in the Nelson Mandela Bay, South Africa. Overall, the findings showed that the educational intervention significantly improved WASH KAP among ECD practitioners. A previous study reported on the positive influence that WASH had in reducing episodes of diarrhoea and acute respiratory infections in 5-year-old children and their families [31]. However, the current study noted a reduction in knowledge on variables which indicated that ‘water containers must always be clean’ and ‘waste can be breeding sites for flies and rodents’. This compares well with findings from a similar study which reported a decline in knowledge levels of some variables post intervention [32]. This suggests the need to ensure that the intervention materials are simple, well-understood and applicable.

The availability of electricity, flush toilets and well-ventilated sanitation areas and separate toilet facilities for staff and children in ECD centres is an important facilitator to enhance WASH status, which in the study setting not all ECD centres had access to. Reports by WHO and UNICEF [3, 6, 13, 30] also reported that lack of access to safe water and adequate sanitation services affects WASH status, which can result in adverse effects in early childhood survival, and is associated with stunting and poor academic performances, productivity, self-esteem, and satisfaction [33, 34]. Additionally, a previous WHO and UNICEF report in 2019 indicated that diarrhoeal and parasitic infections are associated with inadequate access to water, poor sanitation, and unhygienic practices, causing billions of preventable diseases such as diarrhoeal and parasitic diseases leading to the mortality of millions of people especially children less than five years of age [34, 35]. Recommendations therefore need to be made to improve infrastructure for increased access to facilities enhancing access to safe water and sanitation at these ECD centres. Short-term solutions could include mobile toilets, regular cleaning of sanitation areas with bleach water and the heated buckets, as provided to some ECDs in our study without running water, which could be done through public-private partnerships, as recommended elsewhere [36].

In the current study, there was a notable improvement from participants’ KAP responses post intervention on certain items while other items recorded declines in valid responses, which was also found in similar WASH studies [37]. For instance, the variable ‘washing hands every time further wastes the water that the Bay wants us to save’ recorded 76.47% post intervention. This finding indicates that the intervention could have enhanced awareness leading to improved KAP, however, some items may not have been fully addressed by the intervention due to a possible need to extend the intervention duration or strategy. Moreover, overall KAP scores significantly improved and respondents’ practices regarding toilet facilities ventilation status and potties facilities ventilation status showed a statistically significant difference in the WASH knowledge scores, which was confirmed by a previous study by Yates et al. [37] highlighting the KAP variables as significant factors which can be maximized by intervention programs in enhancing the awareness, understanding, attitudes and practices as well as behavioural patterns that affect health outcomes [38]. In order to optimise the impact of educational intervention impact, it is recommended that the intervention should be adapted to enhance emphasis on items that showed a decline in correct responses, such as the attitude related items (e.g., ‘caregivers should lead by example by keeping good hygiene’), the importance of hand washing and the practice related item (e.g., cleaning of classroom areas), as recommended in the study by Yates et al. [37]. Furthermore, there is a need for the implementation of a sustainable and integrated intervention measures strategically designed and monitored to enhance KAP via human capacity development, which fosters acceptability and enhances health outcomes. A previous pre-post intervention study by Rassi et al. [32] associated the enhancement in participants general awareness of Schistosomiasis to regular mass drug administration campaigns over a number of years [32]. The study of Rassi et al. [32] further indicated that approximately 14 months of implementing an intervention may still be grossly inadequate to influence a behavioural change. The aforementioned was further alluded to in a previous study by Michie et al. [39] which emphasised that improved knowledge does not automatically translate to enhanced practices [39] or behavioural modifications. Further research would therefore be required to possibly determine the effective intervention dose, percentage of the population required to actively involved in the project as well as the frequency and duration of the engagement programs for enhanced uptake. As indicated by Michie et al. [39], this strategy is envisaged to create a platform for a community-owned intervention which has the potential to enhance community participation as well as correct misconceptions which negatively impact sustainable behavioural change.

### Study limitations

The study noted some limitations such as confinement of the study to a particular municipality. Inclusion of other municipalities in the province or other districts may have yielded possibly different results. Since the participants in the current study were only ECD practitioners, it could be possible that if the inclusion criteria were widened to include mothers and children in the ECD centres, a different result may have been obtained. Furthermore, we utilised a quantitative research design to examine the relationship between variables which may inadvertently omit evaluation and inclusion of variables that could be unravelled by assessing participants’ perceptions and experiences qualitatively. The low response rate/small sample size which limits generalisation or extrapolation of this study can be improved on in future studies by either increasing the number of pre- and post- intervention participants or the data collection periods. Moreover, we cannot rule out the possibility of bias attributable to social desirability which might have influenced the participants’ responses to the survey questions to what the ‘popular opinion’ or socially desirable response may be. This is particularly possible in the post-intervention responses since the participants were now exposed to intervention strategies designed to enhance KAP and associated behavioural outcomes. Finally, this study was limited to WASH KAP only and further studies should be conducted to evaluate the impact on disease outbreaks common among children aged 5 years and younger at ECD centres.

## Conclusion

The results from this study revealed that the educational intervention significantly improved WASH KAP among ECD practitioners. Toilet facilities ventilation status positively influenced WASH knowledge scores and potties facilities ventilation status significantly improved WASH attitude scores. Bush/field (no facility) and plastic potties usage significantly influenced practice scores. Findings from the study highlights the degree to which WASH interventions could enhance hygiene knowledge, attitudes and practices that promotes healthy outcomes which could inform practitioners and policymakers concerned with initiating school-based WASH programs and community outreach activities. These results could be used as the basis for the development of a national policy or strategic plan regarding WASH, and strategies to enhance behavioural modifications for enhanced acceptability and sustainability of WASH intervention strategies. It is becoming increasingly clear that unhygienic WASH practices are positively associated with disease transmission and childhood stunting. There is therefore an urgent need to consider the inclusion of WASH knowledge and practices in the curriculum of ECD centres to prevent and control disease transmissions and exposures in early childhood.

## Supporting information

S1 Data (XLSX)
